# Supplementary material for: Assessment of knowledge on self-management and level of asthma control among patients attending a tertiary care center in Nepal: A cross-sectional study
Source: PLOS Glob Public Health. 2026 May 29;6(5):e0006563. doi: 10.1371/journal.pgph.0006563 (PMC13221001; doi:10.1371/journal.pgph.0006563)
Supplement: S1 File — a. The first page consists of the informed consent form. b. Second page consists of the socio demographic characteristics. c. Third page consists of the 5 items of the Asthma Control Test. d. The remaining ones consists of the 16 items of Asthma Self-Management Questionnaire. (DOCX) [file pgph.0006563.s001.docx]

**Informed Consent form**

I have been informed about the research project titled “Assessment of Knowledge on Self-Management and Level of Asthma Controls among Patients Attending a Tertiary Care Center in Nepal: A Cross-Sectional Study” being carried out by the Principal Investigator Dr. Swojay Maharjan.

I understand that my participation in this research is voluntary and that I can stop from the research process at any moment if I so choose. I've been told that my legal rights and the services I receive won't be impacted, and I won't be required to provide an explanation. I understand that no personally identifiable information about me will be published in the report of this research or in related publications. In light of all of this, I sign this informed consent form and willingly consent to take part in this research project.

Participant’s Signature:

Participant’s Name:

Thumb print

Right Left

Date 2024/ /

**Questionnaire**

**SBHF number:**

**Contact number:**

**Age:**

**Gender:**

**Occupation:**

1. **Army personnel**
2. **Housewife**
3. **Small business persons**
4. **Farmer**
5. **Others (retired, teachers, workers, so on)**

**Level of education:**

1. **Illiterate**
2. **Literate**
3. **Secondary**
4. **Higher secondary**
5. **University**

**Duration of asthma in years**

**Family history of asthma: 1. Present 2. Absent**

**Are any allergen known to you? 1. Yes 2. No**

**If Yes then please specify the allergen…..**

**Have you smoked cigarette in the past? 1. Yes 2. No**

**If Yes then specify the duration and number of cigarettes smoked per day….**

**Do you spend time with pets at work or at your home? 1. Yes 2. No**

**Do you dwell in a dampy, dusty room? 1. Yes 2. No**

**Have you experienced asthma exacerbation in the past 1 year? 1. Yes 2. No**

**Comorbidities:**

1. **DM**
2. **HTN**
3. **Hypothyroidism**
4. **Others:**

**Asthma Control Test**

1. **During the last 4 weeks, how much time has your asthma kept you from getting as much done at work, school or home?**
   1. **All the time**
   2. **Most of the time**
   3. **Some of the time**
   4. **A little of the time**
   5. **None of the time**
2. **During the past 4 weeks, how often have you had shortness of breath?**
   1. **More than once a day**
   2. **Once a day**
   3. **3-6 times a week**
   4. **Once or twice a week**
   5. **Not at all**
3. **During the past 4 weeks, how often did your asthma symptoms (wheezing, coughing, shortness of breath, chest tightness or pain) wake you up at night or earlier than usual in the morning?**
   1. **4 or more nights a week**
   2. **2 – 3 nights a week**
   3. **Once a week**
   4. **Once or twice**
   5. **Not at all**
4. **During the past 4 weeks, how often have you used your rescue inhaler or nebulizer medication (such as Salbutamol)?**
   1. **3 or more times per day**
   2. **1 or 2 times per day**
   3. **2 or 3 times per week**
   4. **Once a week or less**
   5. **Not at all**
5. **How would you rate your asthma control during the past 4 weeks?**
   1. **Not controlled at all**
   2. **Poorly controlled**
   3. **Somewhat controlled**
   4. **Well controlled**
   5. **Completely controlled**

**ASTHMA SELF-MANAGEMENT QUESTIONNAIRE**

**Please circle the letter that corresponds to your answer for each question.**

1. **A main method to prevent asthma flare-ups is to...**
   1. **take medicines before meals**
   2. **take steroids in pill form**
   3. **get a flu vaccine**
   4. **go to the emergency room at the first sign of symptoms**
   5. **I don't know**
2. **Taking the prescribed two puffs of your inhaler two times a day...**
   1. **is the same as taking one puff four times a day**
   2. **is the same as taking four puffs once a day**
   3. **can be arranged in any way as long as you take a total of four puffs a day**
   4. **is not the same as any other regimen**
   5. **I don't know**
3. **If you are not having asthma symptoms...**
   1. **your lungs are not sensitive to irritants**
   2. **it is OK to skip some doses of medicine**
   3. **you should still avoid triggers**
   4. **you are probably cured of asthma**
   5. **I don't know**
4. **Maintenance medicines...**
   1. **help prevent future symptoms**
   2. **don't need to be taken every day**
   3. **make you breathe better right after you take them**
   4. **can only be taken in pill form**
   5. **I don't know**
5. **The correct way to use a peak flow meter is to...**
   1. **take a deep breath and then blow into the mouthpiece slowly**
   2. **start exhaling and then put the mouth- piece in your mouth**
   3. **put the mouthpiece in your mouth and then inhale and exhale**
   4. **take a deep breath then blow into the mouthpiece as fast as you can**
   5. **I don't know**
6. **Rescue medicines...**
   1. **should not be taken more than three or four times a day**
   2. **help prevent future flare-ups**
   3. **have no side effects**
   4. **do not cause you to become tolerant to medicine**
   5. **I don't know**
7. **When using your inhaler, you should...**
   1. **take shallow breaths**
   2. **inhale quickly**
   3. **inhale slowly**
   4. **press your inhaler several times while you are inhaling**
   5. **I don't know**
8. **After you have used your inhaler, you should...**
   1. **hold your breath for several seconds**
   2. **take the second puff as soon as possible after the first puff**
   3. **keep taking puffs until you feel better**
   4. **wash the inhaler in a tub of water**
   5. **I don't know**
9. **If you are having symptoms and don't know why, the first thing you should do is...**
   1. **take some doses of steroid medicine**
   2. **call your doctor**
   3. **count how fast you are breathing**
   4. **change your immediate environment**
   5. **I don't know**
10. **Taking more rescue medicines than prescribed...**
    1. **is really not harmful**
    2. **is a good way to manage symptoms caused by exercise**
    3. **may mean you can take less maintenance medicine**
    4. **may mean you need more maintenance medicine**
    5. **I don't know**
11. **The benefit of using a peak flow meter every day is...**
    1. **you can detect small changes in lung function even before symptoms start**
    2. **it can tell you when you can decrease your medicines**
    3. **you can see how well you can inhale**
    4. **you can have a way to compare yourself to other people with asthma**
    5. **I don't know**
12. **For people with asthma, exercise...**
    1. **is something that should not be done regularly**
    2. **can help improve breathing capacity**
    3. **is only good if done for at least 30 minutes at a time**
    4. **can trigger symptoms because the lungs are not taking in enough oxygen**
    5. **I don't know**
13. **Asthma can be cured by...**
    1. **taking daily medicine**
    2. **avoiding triggers, such as dust and cigarette smoke**
    3. **using a peak flow meter**
    4. **there is no known cure for asthma**
    5. **I don't know**
14. **Asthma flare-ups...**
    1. **usually occur suddenly without warning**
    2. **can occur when several minor triggers come together**
    3. **cannot be triggered by strong emotions**
    4. **always cause wheezing**
    5. **I don't know**
15. **If you are prescribed a seven-day course of steroid pills...**
    1. **you don't have to avoid triggers while you are taking the pills**
    2. **your symptoms can't get worse while you are taking the pills**
    3. **you don't need to use your peak flow meter while you are taking the pills**
    4. **you should finish the prescription even if you feel better after several doses**
    5. **I don't know**
16. **Which of the following can help control asthma?**
    1. **reducing stress levels**
    2. **drinking plenty of water to stay hydrated**
    3. **avoiding foods with sulfites, such as dried fruits and wine**
    4. **all of the above**
    5. **I don't know**
