# Supplementary material for: Assessment of knowledge on self-management and level of asthma control among patients attending a tertiary care center in Nepal: A cross-sectional study
Source: PLOS Glob Public Health. 2026 May 29;6(5):e0006563. doi: 10.1371/journal.pgph.0006563 (PMC13221001; doi:10.1371/journal.pgph.0006563)
Supplement: Attachment — Submitted filename: Comments for Manuscript.pdf [file pgph.0006563.s004.pdf]

Comments

Section	comment
Methods	
Study Design	"Assessing asthma self-management using standardized methods and connecting it to disease control makes sense in a Low-and-Middle-Income-Country (LMIC) like Nepal where the doctor-to-patient ratio is subpar and there is a significant intellectual divide between them."<br>Provide reference of this line
Study Sample	"A nonprobability convenient sampling method was used – where the convenience to the researchers was in the process of data collection, which was done at researchers' appropriate time."<br>Make this sentence grammatically correct
Study Instrument	Author written both ACQ and ASMQ were translated but did not report about validity and reliability check of translated instrument.
Results	Some sociodemographic characteristics like "time spent with pets" and "damp and dusty room" does not make much sense for checking association with asthma self-management knowledge Score (ASMQ), but this could make more sense if author check these variable association with asthma control test score as pet and damp, dusty room can affect Asthma control status. (But since author did not check association between sociodemographic and asthma control score, this is just suggestion)<br>Any particular reason for not checking association between age and ASMQ.
Discussion	In the discussion the 2nd and 3rd paragraph is simply the replication of results. Author did not related these findings with existing literature, so it is suggested for author to related it to existing literature.<br>Author could also include asthma control status and compare it with existing literature i.e. how many are having partially controlled, uncontrolled asthma etc.
Conclusion	"We found that patients' attitudes and practices were closely linked to their knowledge of asthma self-management".<br>The study measure Asthma control and self-management knowledge. So how author concluded regarding attitude?
