# Supplementary material for: Assessment of knowledge on self-management and level of asthma control among patients attending a tertiary care center in Nepal: A cross-sectional study
Source: PLOS Glob Public Health. 2026 May 29;6(5):e0006563. doi: 10.1371/journal.pgph.0006563 (PMC13221001; doi:10.1371/journal.pgph.0006563)
Supplement: Attachment — Submitted filename: Response to Reviewers.docx [file pgph.0006563.s005.docx]

25^th^ November, 2025

Katrien G. Janin, PhD

Staff Editor

PLOS Global Public Health

Subject: Rebuttal letter responding the editor’s and reviewer’s comments.

Respected Sir/Ma’am,

I would like to thank the editorial team and the reviewers for the opportunity to revise the manuscript such that it met the standards and the implication or impact it would create to the evidence based practice.

Here are our responses to the comments

**Journal requirements**

1. **In the online submission form, you indicated that the data that support…**

We have now uploaded the de-identified raw dataset (including item-level ASMQ and ACT scores) as a supplementary file in SPSS format and agree to make it freely accessible in accordance with PLOS data-sharing policies.

2. **Please amend your detailed Financial Disclosure statement. This is published..**

The Financial Disclosure statement has been revised under the “Source of Funding” section to read: *“The authors received no specific funding for this work.”*

3. **We have noticed that you have uploaded Supporting information files, but you have…**

A full list of legends for all supplementary files has been added following the reference list.

4. **If the reviewer comments include a recommendation to cite specific previously published works…**

We reviewed all recommended references and incorporated the relevant ones into the manuscript.

**Reviewer #1 comments**

(Page and line numbers refer to the revised manuscript with track changes.)*

A. **Introduction**

Relevant studies have been incorporated in page 2 lines= 63 to 71 and the practical significance of the research has been emphasized, particularly in the conclusion in page 17.

B. **Scientific and Methodological Rigor**

(Page 14 and 15, Lines )* We have expanded the Discussion to clearly acknowledge the limitations associated with convenience sampling and the implications for generalizability. Overstatements suggesting causality have been revised to reflect associations only.

C. **Statistical Analysis**

(Page 6, Lines 203, 206, 215)* A more comprehensive justification for the use of non‑parametric tests has been added, along with a clearer explanation of how normality was assessed. (Page 12, line 264 to 268) * Multiple-comparison adjustments are now explained in the Results section before Table 3.

Regarding effect sizes:
For dichotomous variables, effect sizes are reported as rank biserial correlations (r₍rb₎). For variables with more than two categories (occupation and educational level), effect sizes are reported using Kruskal–Wallis eta-squared (η²ₕ), which represents the proportion of variance in ASMQ scores attributable to group differences.

D. **Data Availability**

The complete de-identified dataset is now provided as supplementary material per PLOS requirements.

E. **Language and Clarity**

We undertook a comprehensive language revision to address grammatical inconsistencies, redundancies, and unclear phrasing. Terminology has been standardized (e.g., “convenience sampling method,” removal of unclear statements).

F. **Presentation of Results**

Pages 8 - 10)* Table 2 has been reorganized to group similar misconceptions for improved readability. Figure 2 has been updated with clearer labeling and legends.

G. **Discussion and Literature Contextualization**

The Discussion has been strengthened by:

- Highlighting implications for low‑ and middle‑income countries, particularly Nepal.
- Explicitly addressing limitations such as self-reported data and selection bias.
- Integrating more recent literature to contextualize findings.

**Reviewer #2**

(Page and line numbers refer to the revised manuscript with track changes.)*

**P6**

“Refusal to Participate” was removed from the Study Sample Section.

**P7**

**A little too much information in the Statistical Analysis section eg detail on checking…**

**(Page 6, line 203 *)**Excessive procedural detail about normality testing has been condensed. A concise statement now reads: “Since the assumption of normality for ASMQ scores was not satisfied for all combinations of independent variables, non-parametric tests were applied.”

**In the study instruments section, please provide some more detail about questionnaire..**

(**Page 5, line 150 to 177)**Additional detail has been added about the ASMQ and ACT questionnaires, including interpretation (e.g., higher ASMQ scores indicate greater knowledge) and administration procedures.

**P13**

**Table 4: It is unclear as to which comparison the p of 0.011refers to**

**(Page 14, line 287)** We clarified that the p-value of 0.011 represents the Kruskal–Wallis test comparing ASMQ scores across the three ACT subgroups.

**P14**

**The current content of the Discussion could be trimmed a little..**

The Discussion has been streamlined to reduce redundancies and now includes a new section outlining potential strategies for improving asthma knowledge and their possible effects on asthma control.

We greatly appreciate the reviewers’ insightful comments, which have significantly strengthened our manuscript. We hope the revisions meet the journal’s expectations and we remain happy to address any further queries.

Thank you for your time and consideration.

Sincerely,
Kushal Shrestha **(Corresponding Author)**
On behalf of all co-authors
