# Supplementary material for: Assessment of knowledge on self-management and level of asthma control among patients attending a tertiary care center in Nepal: A cross-sectional study
Source: PLOS Glob Public Health. 2026 May 29;6(5):e0006563. doi: 10.1371/journal.pgph.0006563 (PMC13221001; doi:10.1371/journal.pgph.0006563)
Supplement: Attachment — Submitted filename: Response_to_Reviewers_auresp_2.docx [file pgph.0006563.s006.docx]

23^rd^ April, 2026

Alejandro Torrado Pacheco, PhD

Staff Editor

PLOS Global Public Health

Subject: Rebuttal letter responding the reviewer’s comments.

Thank you for your thorough review and constructive suggestions. We have carefully addressed each of your comments and revised the manuscript accordingly. Our responses are outlined below:

(Page and line numbers refer to the revised manuscript with track changes.)*

**1. Methods – Study Design (Reference required)**
We appreciate this observation. The statement regarding challenges in LMIC settings, including limited doctor-to-patient ratio and gaps in health literacy, has now been supported with the reference of a systematic review by Salim et al (Page 3, Line 95).

**2. Study Sample – Sentence correction**
The sentence has been revised for clarity and grammatical accuracy as follows (Page 3, Lines 108- 110) :
“A non-probability convenience sampling method was employed, wherein participants were selected based on accessibility, and data collection was conducted at times convenient to the researchers.”

**3. Study Instruments – Validity and reliability of translated tools**
Thank you for highlighting this important point. We have now clarified that the ASMQ and ACT were translated using a standard forward-backward translation procedure which was reviewed by two pulmonologists and one public health expert (Page 4, Lines 121- 123). The Cronbach’s alpha for ASMQ was 0.513 and ACT was 0.554.

**4. Results – Inclusion of variables such as pets and damp/dusty environment**
We appreciate this insightful suggestion. These variables were included as part of a broader assessment of environmental and lifestyle factors. While they may not directly influence self-management knowledge (ASMQ), we agree that they are more relevant to asthma control outcomes. This has now been acknowledged in the discussion as a limitation and an area for future research, including potential analysis with asthma control status. (Page 13, Lines 340-342)

**5. Association between age and ASMQ score**
Thank you for this important query. We did perform an analysis to assess the correlation between age and ASMQ score using Spearman’s correlation. However, the result did not provide clinically meaningful insight for interpretation within the scope of this study; therefore, it was not included in the final manuscript.

**6. Discussion – Repetition of results**
We agree with this observation. The second and third paragraphs of the discussion have been revised to reduce redundancy and now better integrate our findings with existing literature, improving the interpretative depth of the manuscript. (Page 12, Lines 263-267, 273-275)

**7. Comparison of asthma control status with existing literature**
Thank you for this valuable suggestion. We have now expanded the discussion to compare the proportion of controlled, partially controlled, and uncontrolled asthma in our study with findings from relevant regional and international studies. (Page 12, Lines 290-297)

**8. Conclusion – Statement on attitudes and practices**
We appreciate this critical observation. As the study did not directly assess attitudes and practices, this statement has been revised. The conclusion now strictly reflects the measured variables—self-management knowledge and asthma control—and their observed association. (Page 14, Lines 348-352)

We sincerely thank you for your insightful comments, which have significantly improved the quality and clarity of our manuscript.

Sincerely,
Kushal Shrestha
On behalf of all authors
